# Monocytes with angiogenic potential are selectively induced by liver resection and accumulate near the site of liver regeneration

**DOI:** 10.1186/s12865-014-0050-3

**Published:** 2014-10-30

**Authors:** Dominic Schauer, Patrick Starlinger, Philipp Zajc, Lejla Alidzanovic, Thomas Maier, Elisabeth Buchberger, Lorand Pop, Birgit Gruenberger, Thomas Gruenberger, Christine Brostjan

**Affiliations:** Department of Surgery, Medical University of Vienna, General Hospital, Waehringer Guertel 18-20, 1090 Vienna, Austria; Department of Internal Medicine, Barmherzige Brueder Hospital, Johannes von Gott Platz 1, 1020 Vienna, Austria

**Keywords:** Monocytes, Liver resection, M-CSF, Colorectal liver metastases, Liver regeneration

## Abstract

**Background:**

Monocytes reportedly contribute to liver regeneration. Three subsets have been identified to date: classical, intermediate, non-classical monocytes. The intermediate population and a subtype expressing TIE2 (TEMs) were suggested to promote angiogenesis. In a clinical setting, we investigated which monocyte subsets are regulated after liver resection and correlate with postoperative liver function.

**Methods:**

In 38 patients monocyte subsets were evaluated in blood and subhepatic wound fluid by flow cytometry before and 1-3 days after resection of colorectal liver metastases. The monocyte-regulating cytokines macrophage colony stimulating factor (M-CSF), transforming growth factor beta 1 (TGFβ1), and angiopoietin 2 (ANG-2) were measured in patient plasma by ELISA. C-reactive protein (CRP) and liver function parameters were retrieved from routine hospital analyses.

**Results:**

On post-operative day (POD) 1 blood monocytes shifted to significantly elevated levels of intermediate monocytes. In wound fluid, a delayed surge in intermediate monocytes was detected by POD 3. Furthermore, TEMs were highly enriched in wound fluid as compared to circulation. CRP and M-CSF levels were substantially increased in patient blood after surgery and correlated significantly with the frequency of intermediate monocytes. In addition, liver function parameters showed a significant association with intermediate monocyte levels on POD 3.

**Conclusions:**

The reportedly pro-angiogenic subsets of monocytes are selectively increased upon liver resection and accumulate next to the site of liver regeneration. As previously proposed by *in vitro* experiments, the release of CRP and M-CSF may trigger the induction of intermediate monocytes. The correlation with liver parameters points to a functional involvement of these monocyte populations in liver regeneration which warrants further investigation.

**Electronic supplementary material:**

The online version of this article (doi:10.1186/s12865-014-0050-3) contains supplementary material, which is available to authorized users.

## Background

Liver resection or ablation remains the only potentially curative option for patients with colorectal liver metastases to date. Factors determining the risk of post-operative complications which may occur in 40-50% of patients have been investigated extensively [1-3]. Generally, morbidity is related to the ability of the remnant liver mass to regenerate and thus fulfil its metabolic functions [4]. Monocytes as central players of the immune system play a pivotal role in liver regeneration. While overall numbers of monocytes increase after surgery, their immune function is partly suppressed [5,6]. Of note, a two-fold increase in monocytes on the first post-operative day has been found predictive in terms of enhanced overall survival after resection of colorectal liver metastases [7].

The complex regulation of monocytes involves an array of proteins including C-reactive protein (CRP), macrophage colony stimulating factor (M-CSF), transforming growth factor beta 1 (TGFβ1) and angiopoietin 2 (ANG-2) [8-10]. These mediators are known to direct the recruitment and differentiation of monocyte populations for functional responses in phagocytosis, antigen presentation, angiogenesis and cytokine release. Furthermore, monocytes that migrate into the tissue develop into macrophages and dendritic cells which shape the local immune response and tissue regeneration [11-13]. The importance of M-CSF and monocyte-derived macrophages in liver regeneration has been investigated in mice genetically lacking functional M-CSF [11]. These mice showed a reduction of Kupffer cells in the liver by 60%. After partial hepatectomy the mice exhibited a significantly reduced proliferation of hepatocytes and a delayed hepatic regeneration. Treatment with recombinant M-CSF resulted in the recovery of Kupffer cell counts and liver regeneration.

An indication as to how M-CSF is induced upon liver damage came from the *in vitro* observation that the acute phase protein CRP may stimulate endothelial cells and macrophages to secrete M-CSF [14]. M-CSF is known to function as a chemoattractant for monocytes to inflamed or injured tissues [15] and can further promote survival and differentiation of monocytes [16,17]. Thus, it was shown that cytokines such as M-CSF and TGFβ1 trigger a phenotypic shift of circulating human monocytes by inducing the expression of CD16, the low-affinity immunoglobulin G receptor involved in antibody-dependent cellular cytotoxicity [8,10].

By differential expression of their receptors CD16 and CD14, the co-receptor for lipopolysaccharide (LPS), human monocytes are classified as CD14++CD16- “classical monocytes”, CD14++CD16+ “intermediate monocytes” and CD14+CD16++ “non-classical monocytes” [18]. Functional and genetic studies have indicated a developmental relationship between these subsets with gradual changes in surface markers during maturation as well as distinct biological properties [19,20]. Classical monocytes which amount to 85% of circulating monocytes, show the highest phagocytosis potential and are potent producers of pro-inflammatory cytokines in response to the bacterial component LPS [19,20]. In contrast, non-classical monocytes, amounting to 10% of total monocytes, exhibit a “patrolling” (crawling) behaviour along vessel walls and react strongly against viruses [21,22]. Intermediate monocytes have been linked to antigen presentation as well as angiogenesis [20]. Thus, factors involved in MHCII complex formation (HLA-DR and CD74) and angiogenic molecules such as TIE2, endoglin and VEGFR2 (vascular endothelial growth factor receptor 2) are expressed at highest levels in the intermediate subset and point to functions in tissue remodelling [9,20].

A rare subset of monocytes is known to express TIE2, the receptor for angiopoietins, and is therefore termed TIE2 expressing monocytes (TEMs) [23]. With respect to the official classification of monocytes, TIE2 expression is predominantly but not exclusively detected on intermediate monocytes [9,20]. TEMs were found to migrate to angiogenic sites in tumours potentially involving chemotaxis in response to ANG-2 [23,24]. They may stimulate angiogenesis by the paracrine secretion of growth factors such as VEGF and basic fibroblast growth factor [24].

While the majority of functional studies have been conducted in mouse models, substantial evidence has additionally been gathered in the human setting demonstrating that human intermediate monocytes and TEMs carry angiogenic markers and have angiogenic properties [9,20,25]. Thus, endothelial cell sprouting and tube formation were more potently induced by human TEMs than by TIE2-negative monocytes [25], and intermediate as opposed to classical and non-classical monocytes were described as the subset with distinct tube forming capability [20].

In contrast to studies performed on cancer patients, little is known about the regulation and contribution of pro-angiogenic monocyte subsets in the context of clinical liver resection and regeneration. Since surgery triggers the release of CRP from the liver and as a consequence may raise the levels of circulating M-CSF, a shift in monocytes towards CD16 positive subsets and their functional properties is conceivable. Given the pro-angiogenic potential of CD16+ intermediate monocytes and TEMs, we hypothesized that they are induced by liver surgery and subsequently localize at the resection site to promote tissue regeneration. Conversely, failure of the immune system to recruit the reportedly pro-angiogenic TEMs and intermediate monocytes might result in adverse outcome after surgery.

To address this subject, we conducted a study on 38 patients undergoing resection of colorectal liver metastases. We examined postoperative changes in the monocyte profile of patient blood as well as in wound fluid of subhepatic drainages. Alterations in monocyte subsets were investigated for a potential correlation with circulating levels of monocyte regulating cytokines including CRP, M-CSF, TGFβ1 and ANG-2. Furthermore, monocyte populations and related cytokines were assessed for a potential association with clinical parameters of liver function and injury after hepatic resection.

## Methods

### Patient collective

We analysed a total of 38 patients with liver metastases from colorectal cancer undergoing hepatic resection at the Department of Surgery, Medical University of Vienna between 2007 and 2011. Prior to liver surgery all patients underwent chemotherapy with a standard combination, mostly including bevacizumab. The majority of patients had their primary tumour resected before diagnosis and treatment of metastases. Patients with pre-existing liver disease were excluded from the study. The patient characteristics, their pre-operative variables of liver function and type of hepatic resection are summarized in Table 1. Liver resections were classified in minor (≤3 segments) and major (>3 segments) hepatectomy. Comparably, a sex- and age-matched control collective of 32 healthy individuals was included in the study. Analyses of patient samples were approved by the Institutional Ethics Committee (#300/2006, #437/2006, #791/2010). All patients gave written informed consent.

**Table 1:** **Patient demographics**

**Parameter**	**N (%)**
Sex	
Male	24 (63%)
Female	14 (37%)
Site of primary tumour	
Colon	23 (61%)
Rectum	15 (39%)
Concomitant primary resection	7 (18%)
Type of hepatic resection	
Major	20 (53%)
Minor	18 (47%)
Preoperative parameters of liver function	**Median (Range)**
Bilirubin (mg/dl)	0.64 (0.35-1.71)
ALAT (U/l)	22 (9–346)
ASAT (U/l)	28 (8–404)
gGT (U/l)	35 (13–266)
PT (%)	107 (45–135)
Age (years)	65 (42–80)

gGT: gamma-glutamyltransferase, ALAT: alanine aminotransferase, ASAT: aspartate aminotransferase, PT: prothrombin time.

### Sample collection

Blood was drawn before surgery (pre-OP) prior to the patients’ transfer to the operating room and on post-operative day (POD) 1 and 3. Furthermore, in a subset of 12 patients wound fluid was additionally collected on POD 1 and POD 3. Surgical drainage catheters were placed intraabdominally directly subhepatic, to collect subhepatic wound exudate (SHW) close to the site of liver regeneration.

### Analysis of monocyte populations

Blood was drawn into EDTA (ethylenediamine tetraacetic acid) containing tubes and processed at room temperature. Surface expression of CD14, CD16 and TIE2 was measured applying direct immunofluorescence staining followed by a lyse-no-wash procedure. In brief, 100 μl of whole blood were incubated with the following antibodies at saturating concentrations for 20 minutes: CD14-FITC (Becton-Dickinson, San Jose, CA, USA), CD16-PC5 (Beckman Coulter, Fullerton, CA, USA) and TIE2-PE (R&D Systems, Inc., Minneapolis, MN, USA). To eliminate erythrocytes the Versa Lyse solution (Beckman Coulter) was added for 20 minutes. Wound exudate was aspirated from drainage bags and transferred into an EDTA containing tube to allow for a comparable mode of sample processing and immunostaining as described for blood samples.

Flow cytometry was immediately performed with an FC500 cytometer (Beckman Coulter). Fluorescence gating parameters were established using antibody isotype controls, and values above the 99% negative staining threshold were considered positive. A total of 300.000 leukocytes were measured. Analysis of flow cytometry data was performed with Kaluza software (Beckman Coulter) and the gating strategy is documented in the Additional file 1. Intermediate monocytes (CD14++CD16+) were identified by high-level expression of CD14 and were further distinguished from classical monocytes (CD14++CD16-) by their co-expression of CD16. Numbers of intermediate and classical monocytes were combined to yield the total CD14++ monocyte count. TEMs were identified by the concomitant expression of CD14 and TIE2 (CD14++TIE2+ cells). Data are given in % frequency of total CD14++ monocytes.

### Analysis of soluble blood parameters

For plasma preparation, blood was drawn into pre-chilled tubes containing CTAD (citrate, theophylline, adenosine and dipyridamole) and was processed on ice within 30 minutes as we have previously described [26]. Plasma samples were stored in aliquots at −80°C until further analysis. For measurement of ANG-2, M-CSF (R&D Systems, Inc., Minneapolis, MN, USA) and TGFβ1 (eBioscience, San Diego, CA, USA) commercially available ELISA kits were applied according to the manufacturers’ instructions. As the levels of active, unbound TGFβ1 were below the detection limit in CTAD plasma, total TGFβ1 was measured after acid-activation of plasma samples according to the manufacturer’s protocol. Thus, TGFβ1 values are given as the concentration of total protein including both, the active, freely circulating and the inactive, latency-associated peptide bound form of TGFβ1. Serum CRP levels and parameters of liver function or injury were available from routine hospital evaluation.

### Statistical analysis

Statistical calculations were based on non-parametric tests using SPSS software version 20 (SPSS Inc., Chicago, IL, USA). Due to the challenges in collecting samples in a clinical setting, the available number of measured parameters varied with time points. Please note that statistical tests for paired samples were conducted, i.e., differences between time points were calculated using the Wilcoxon Test. Correlations between the investigated parameters were determined by Spearman’s rank correlation coefficient. The reported p-values were results of two-sided tests. P-values <0.05 were considered statistically significant. As the study design was explorative, no corrections for multiple testing were performed.

## Results

### Liver resection triggers an increase in intermediate monocytes in patient blood

**Figure 1:**
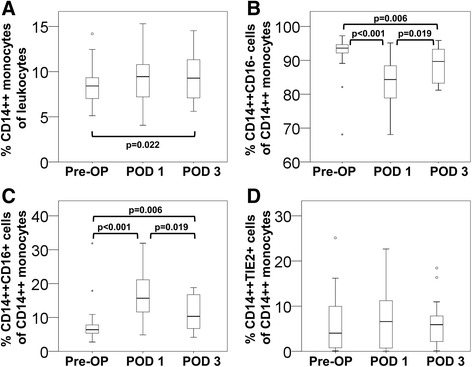
**Perioperative changes in circulating monocyte populations.** Monocyte subsets were determined by flow cytometry in blood samples retrieved from patients undergoing liver resection for colorectal liver metastases. The frequency of total CD14++ monocytes **(A)** was compared to the distribution of classical CD14++CD16- monocytes **(B)**, intermediate CD14++CD16+ monocytes **(C)**, and CD14++TIE2+ TEMs **(D)** prior to surgery (Pre-OP) and on post-operative days 1 (POD 1) and 3 (POD 3).

We applied triple immunostaining and flow cytometry to determine the frequency of monocyte populations in human blood. When patients prior to surgery were compared to a sex-matched (18 male, 14 female) and age-matched (median age 56, range 42–74) control collective of 32 healthy individuals, the distribution of monocyte subsets did not differ significantly (Additional file 2). Perioperative changes of monocyte subsets were then monitored in our collective of patients who underwent surgery for colorectal liver metastases. When determining total CD14++ monocytes in % of all leukocytes, there was no significant change within the first 24 hours and only a moderate increase (p = 0.022, N = 17) on post-operative day 3 (Figure 1A). However, when we evaluated the distribution of classical (CD14++CD16-) and intermediate (CD14++CD16+) subsets within the monocyte population (Figure 1B/C), we found a pronounced decrease in the classical and a substantial increase in the intermediate compartment on POD 1 (p < 0.001, N = 24). While intermediate monocytes ranged at 6% pre-OP, median levels were elevated to 16% after surgery. From POD 1 to POD 3, this trend was reversed (p = 0.019, N = 21) indicating a phase of recovery; but subset distribution did not reach preoperative values (p = 0.006, N = 17). With respect to circulating TIE2 expressing monocytes (CD14++TIE2+), we did not record any surgery-induced alterations (Figure 1D).

### Intermediate monocytes display a delayed increase in wound fluid compared to blood

**Figure 2:**
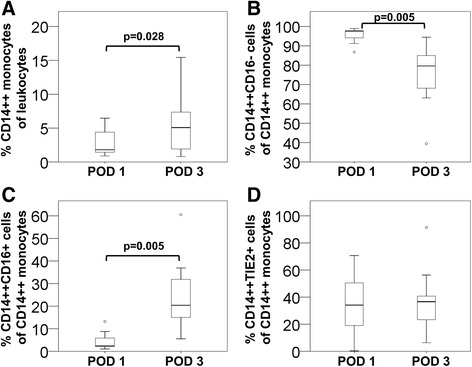
**Distribution of monocyte subsets in subhepatic wound fluid.** Subhepatic wound fluid was collected on POD 1 and POD 3, and the following monocyte subsets were evaluated by flow cytometry: **(A)** total CD14++ monocytes, **(B)** classical CD14++CD16- monocytes, **(C)** intermediate CD14++CD16+ monocytes, and **(D)** CD14++TIE2+ TEMs.

When evaluating monocytes at the wound site, we found a moderate accumulation of total CD14++ monocytes from POD 1 to POD 3 (Figure 2A; p = 0.028, N = 10). Classical monocytes were the predominant fraction (98%) in wound exudate on POD 1 (Figure 2B), while median levels of intermediate monocytes rose to 20% by POD 3 (Figure 2C; p = 0.005, N = 10). The percentage of TIE2+ cells within CD14++ monocytes remained constant in wound fluid during the post-operative observation period (Figure 2D).

### TIE2+ monocytes are enriched at the wound site

**Figure 3:**
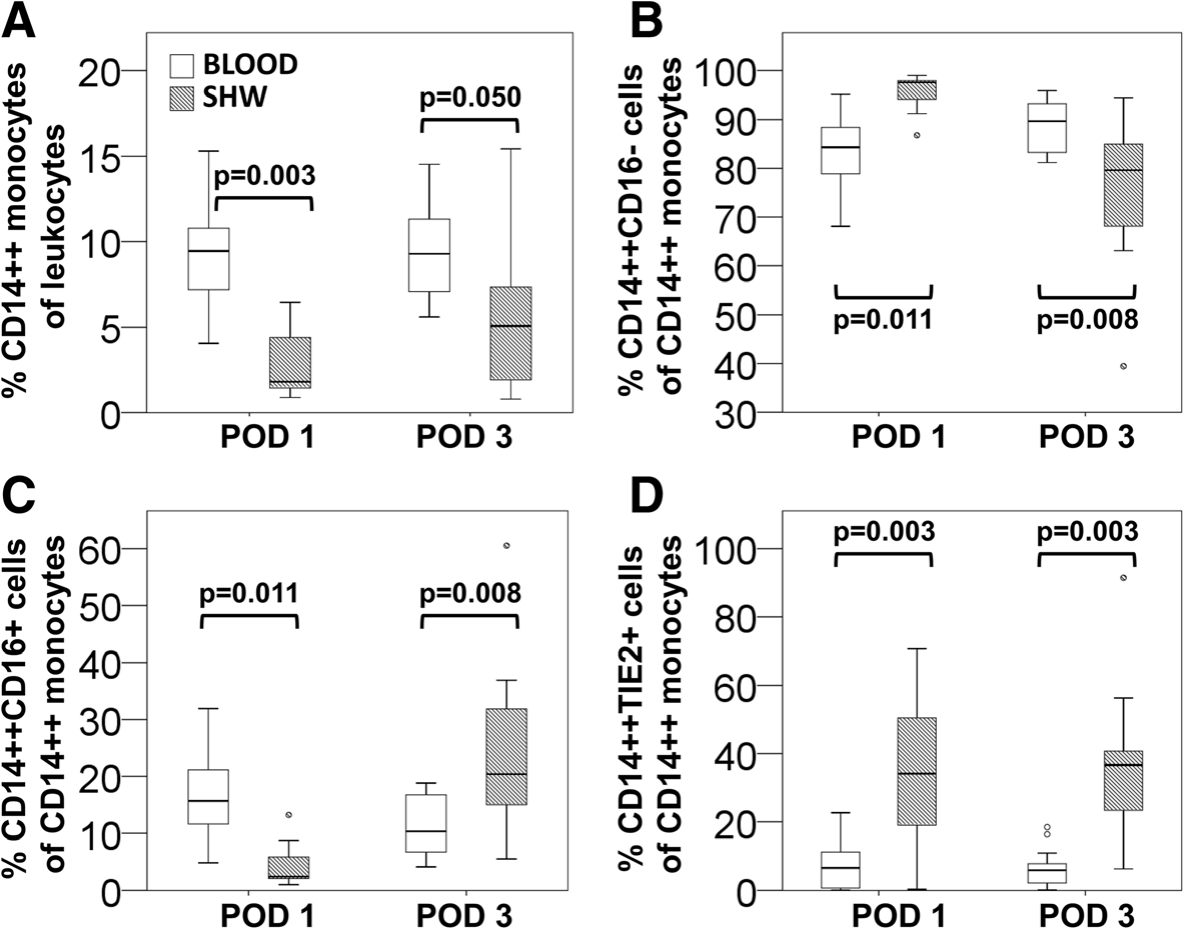
**Comparison of monocyte distribution in blood and wound fluid.** Monocytes in blood and subhepatic wound fluid (SHW) were detected by immunostaining and flow cytometry on POD 1 and POD 3 and were compared with respect to **(A)** the frequency of total CD14++ monocytes and the distribution of classical CD14++CD16- monocytes **(B)**, intermediate CD14++CD16+ monocytes **(C)**, and CD14++TIE2+ TEMs **(D)**.

To evaluate the distribution of monocytes in circulation as opposed to the area close to liver regeneration, we compared the frequency of monocyte subsets in blood and subhepatic wound exudate collected by surgical drainages. Monocytes within total leukocytes (Figure 3A) were less abundant in wound fluid (1.8%) as compared to blood (9.4%) on POD 1 (p = 0.003, N = 11) and increased to 5.1% in wound exudate by POD 3. While peak levels of intermediate monocytes in blood (15.7% of CD14++ cells) were found on POD 1 (Figure 3C), the highest value of intermediate monocytes was recorded on POD 3 at the wound site (20.4% of CD14++ cells). Of interest, the fraction of TIE2+ cells was substantially higher for wound than for blood monocytes (Figure 3D). While the reportedly pro-angiogenic subset of CD14++TIE2+ cells ranged at 6.1% of all CD14++ monocytes in circulation, values reached 35.4% of TIE2 expressing monocytes in post-operative SHW (p = 0.003, N = 11).

### Liver surgery increases blood levels of monocyte regulating cytokines

**Figure 4:**
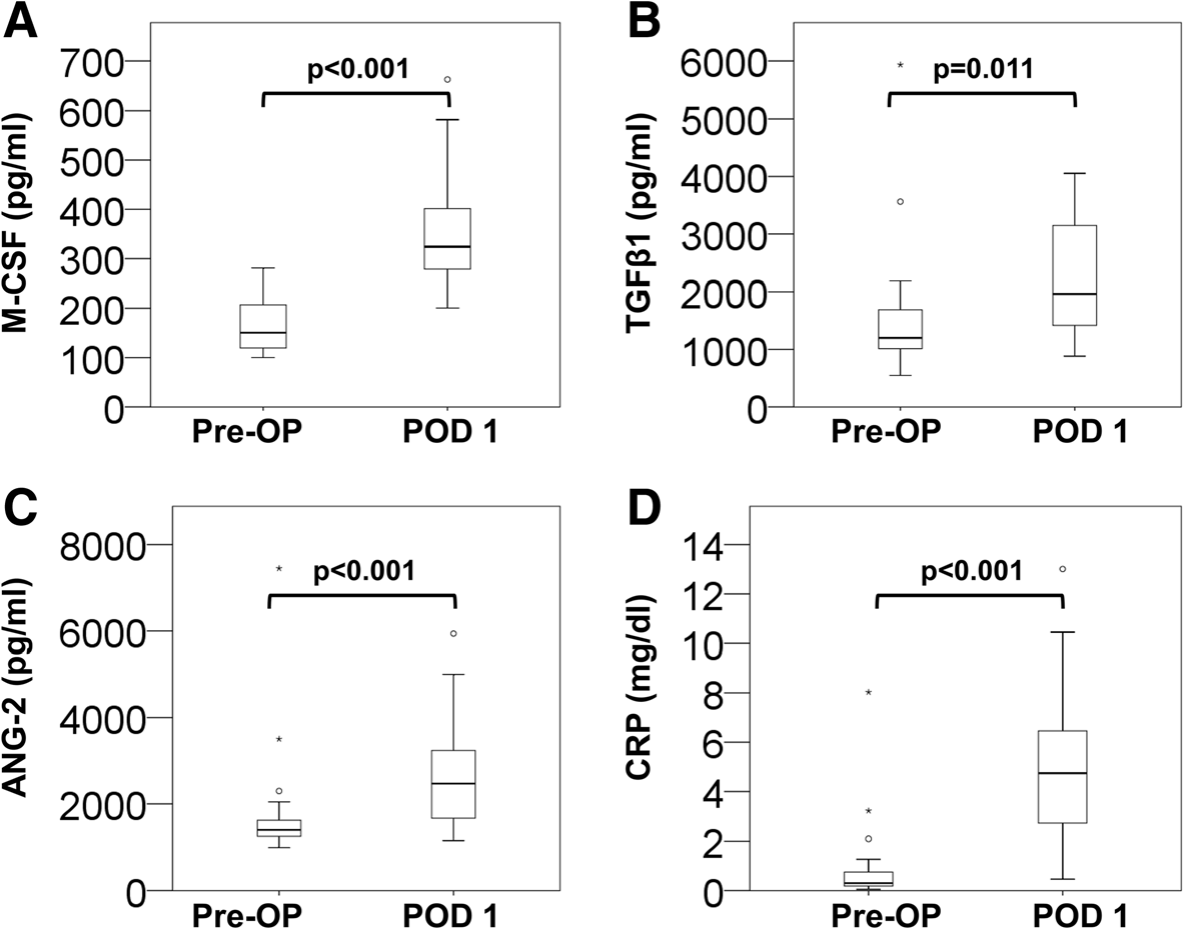
**Perioperative changes in the blood levels of monocyte-regulating cytokines.** M-CSF **(A)**, TGFβ1 **(B)**, and ANG-2 **(C)** were measured by ELISA in plasma samples of patients before (Pre-OP) and after liver surgery (POD 1). Serum concentrations of CRP **(D)** were retrieved from routine hospital analyses.

To identify cytokines that might potentially be involved in regulating the rapid changes of blood monocyte subsets observed on the first day after surgery, we determined the plasma concentrations of M-CSF, TGFβ1, ANG-2 and CRP. We found that median levels of M-CSF (Figure 4A) were more than doubled on POD 1 compared to pre-operative values (p < 0.001, N = 19). Similarly, a significant induction was recorded for total TGFβ1 (Figure 4B; p = 0.011, N = 18) and ANG-2 levels (Figure 4C; p < 0.001, N = 29) on POD 1. Among the investigated blood components, serum CRP levels showed the highest increase (15-fold) on POD 1 (Figure 4D; p < 0.001, N = 35).

### Blood levels of intermediate monocytes correlate with plasma values of M-CSF and CRP

**Figure 5:**
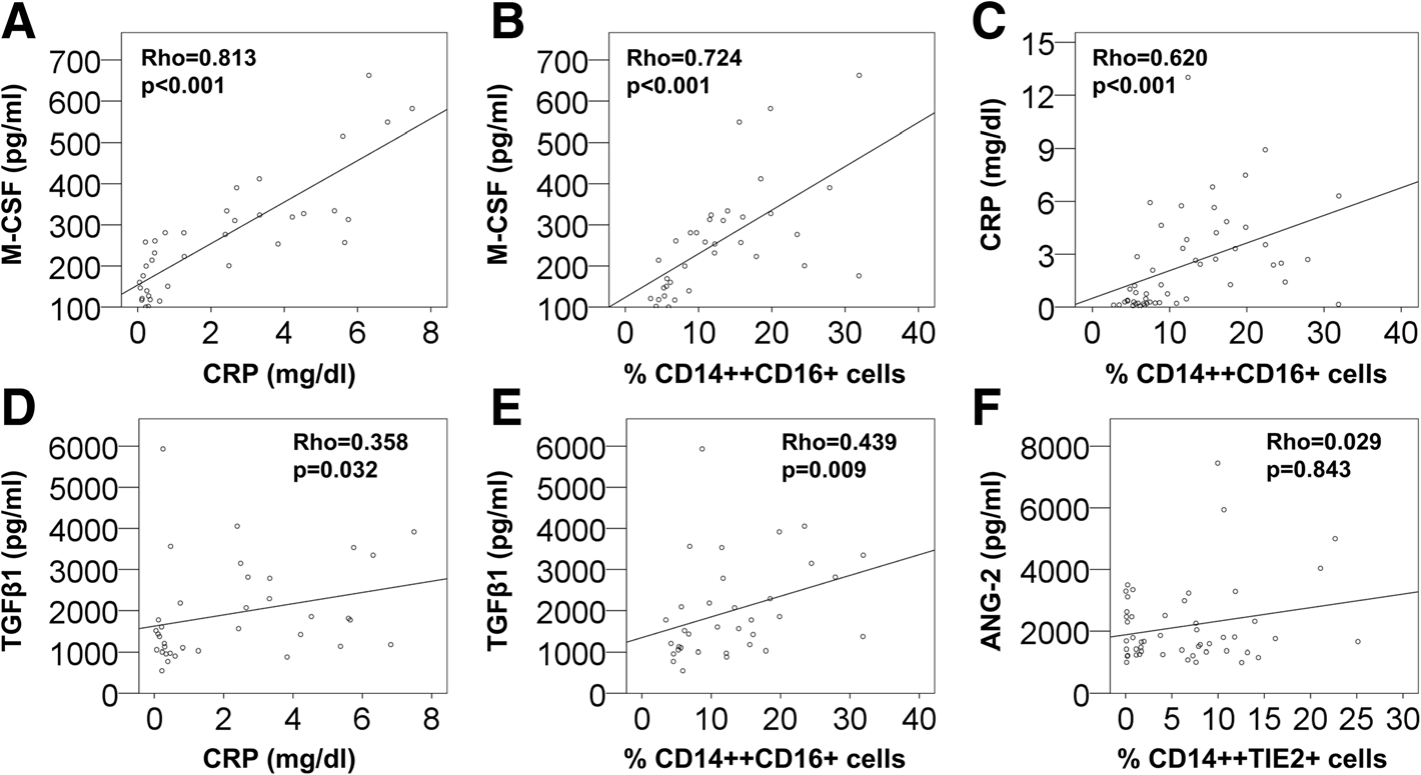
**Correlations between monocyte-regulating cytokines and the intermediate monocyte subset.** Blood levels of CRP and M-CSF **(A)**, and the association of intermediate CD14++CD16+ monocyte counts with M-CSF **(B)** or CRP concentrations **(C)** were evaluated by Spearman’s Rank correlation test. A potential correlation between TGFβ1 and CRP levels **(D)** or TGFβ1 and intermediate CD14++CD16+ monocytes **(E)** was similarly investigated. No correlation was found for perioperative plasma concentrations of ANG-2 and CD14++TIE2+ TEMs **(F)**.

A potential association between the induction of intermediate monocytes and the investigated plasma parameters was subsequently evaluated by Spearman correlation test. CRP blood concentrations correlated positively with M-CSF levels (Figure 5A; Rho = 0.813, p < 0.001, N = 37). For both, CRP (Figure 5C; Rho = 0.620, p < 0.001, N = 52) and M-CSF (Figure 5B; Rho = 0.724, p < 0.001, N = 35) a strong correlation was observed with intermediate monocytes of CD14++ cells, whereas TGFβ1 showed only a weak association with CRP values (Figure 5D; Rho = 0.358, p = 0.032, N = 36) and the frequency of intermediate monocytes (Figure 5E; Rho = 0.439, p = 0.009, N = 34). No significant correlation was detected between TIE2 expressing monocytes and the potentially regulating cytokine ANG-2 (Figure 5F).

### Intermediate monocytes on POD 3 correlate with liver function parameters

**Figure 6:**
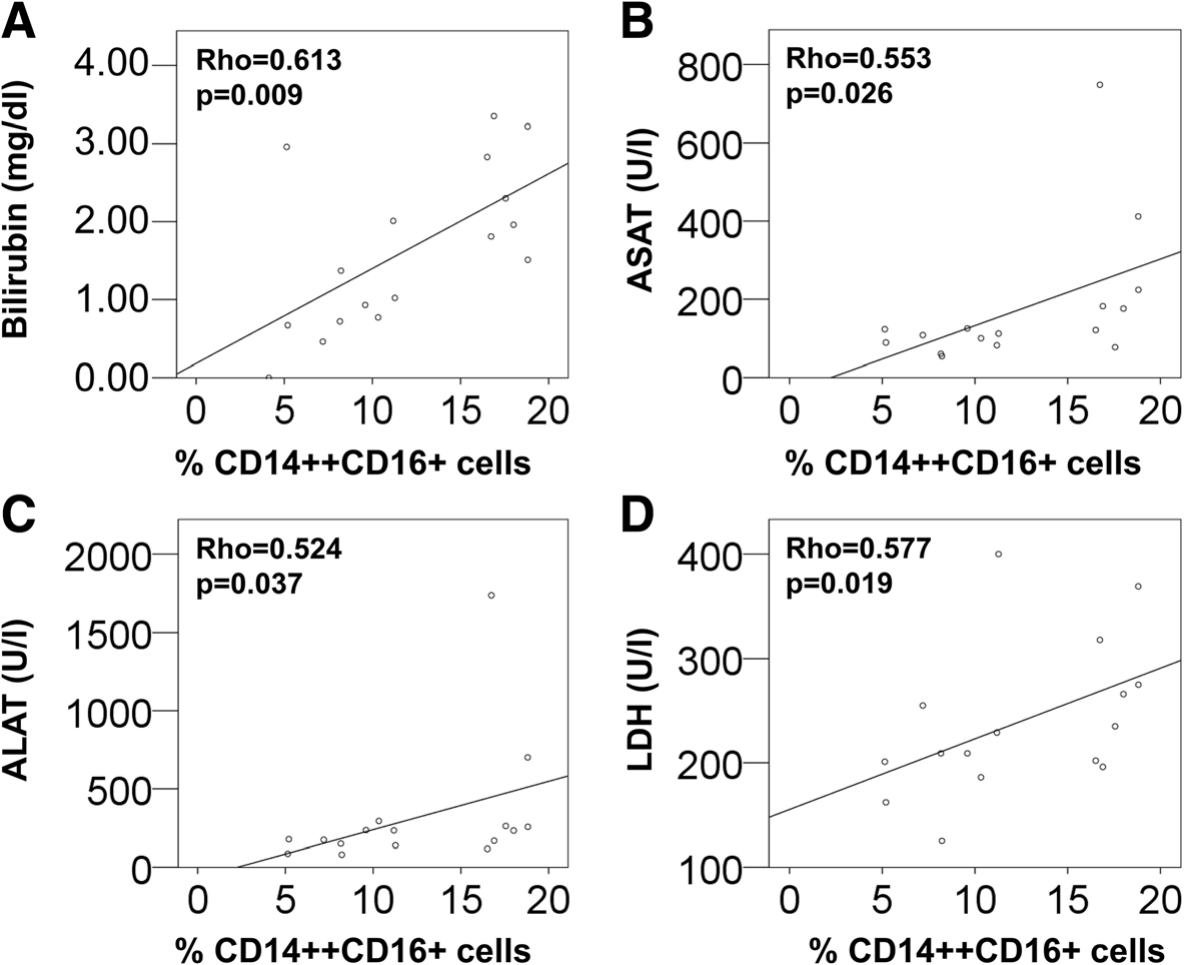
**Correlations between liver function parameters and the intermediate monocyte subset. (A)** Blood levels of bilirubin, **(B)** ASAT, **(C)** ALAT and **(D)** LDH as determined by routine hospital analysis on post-operative days 3–4 were compared to the frequency of intermediate CD14++CD16+ monocytes on POD 3 by Spearman’s Rank correlation test.

To evaluate if altered monocyte distributions after surgery were associated with liver regeneration, clinical parameters of liver function and injury were acquired from routine hospital tests. No correlation was found between intermediate monocytes and liver parameters before surgery or on post-operative day 1. However, significant positive correlations were recorded for the frequency of intermediate monocytes on POD 3 and bilirubin (Figure 6A; Rho = 0.613, p = 0.009, N = 17), aspartate aminotransferase ASAT (Figure 6B; Rho = 0.553, p = 0.026, N = 16), alanine aminotransferase ALAT (Figure 6C; Rho = 0.524, p = 0.037, N = 16), and the cell destruction marker lactate dehydrogenase LDH (Figure 6D; Rho = 0.577, p = 0.019, N = 16) on POD 3–4. Please note that liver parameters on POD 4 were included in case of missing values for POD 3 to increase sample size.

Histological parameters of the resected liver tissue were additionally evaluated (Table 2) to assess whether liver pathologies might be associated with liver function (regeneration) and the intermediate monocyte subset. Neither the stage of fibrosis nor the percentage of steatosis correlated with liver function parameters or intermediate monocytes of POD 3–4. Only one patient presented with sinusoidal obstruction syndrome, while 12 patients (32%) were classified as NASH (non-alcoholic steatohepatitis) or CASH (chemotherapy-associated steatohepatitis). No difference was found in liver function parameters or intermediate monocytes of POD 3–4 between patients with and without steatohepatitis (Additional file 3).

**Table 2:** **Histopathological parameters of resected liver tissue**

**Parameter**	**N (%)**
Fibrosis score*	
0	14 (38%)
1	21 (57%)
2	1 (3%)
3	1 (3%)
4 (cirrhosis)	0 (0%)
ND	1
Steatosis	
<5%	5 (14%)
5-33%	20 (56%)
>33-66%	6 (17%)
>66%	5 (14%)
ND	2
NASH/CASH	
No	25 (68%)
Yes	12 (32%)
ND	1
SOS	
No	36 (97%)
Yes	1 (3%)
ND	1

*Scoring system according to Kleiner et al. [27].

CASH: chemotherapy-associated steatohepatitis, NASH: non-alcoholic steatohepatitis, ND: not determined, SOS: sinusoidal obstruction syndrome.

## Discussion

This study demonstrates that liver resection triggers a strong shift in monocyte subsets and monocyte regulating cytokines. Elevated numbers of the reportedly pro-angiogenic CD16+ intermediate population and decreased numbers of CD16- classical monocytes were detected in patient blood on POD 1 and 3. This rapid increase in circulation was followed by an accumulation of intermediate monocytes in subhepatic wound fluid by POD 3. Comparably, M-CSF, TGFβ1, CRP and ANG-2 increased in patient plasma after surgery; in particular M-CSF and CRP concentrations correlated strongly with the level of intermediate monocytes.

Surgical tissue damage leads to an acute phase response in the liver and hence an increase in CRP release into the blood [28]. Since CRP is known to induce M-CSF expression by endothelial cells [14] and M-CSF up-regulates CD16 expression on human monocytes [8], the observed changes in monocyte phenotype are likely to be triggered as a result of the altered cytokine milieu after surgery. In this regard our findings are in line with a recently published study showing increased values and a correlation of intermediate monocytes, M-CSF and CRP in trauma patients [29].

We would like to note that our study was focused on the analysis of the potentially pro-angiogenic population of CD16+ intermediate monocytes as opposed to the majority of CD16- classical monocytes. With respect to the third subset of CD16++ non-classical monocytes, our detection method was prone to “contamination” with overlapping natural killer cells in flow cytometric measurements [30]. Hence, we generally omitted this subset from our analysis. However, to gain preliminary information whether non-classical monocytes were similarly induced by liver surgery, we devised a modified gating strategy for non-classical monocytes (Additional file 4A): A logarithmic leukocyte density plot was introduced to improve resolution of monocytes, lymphocytes and granulocytes based on light scatter properties. Setting a tight gate on the monocyte population enabled us to minimize “contamination” of non-classical monocytes by CD16++ natural killer cells or granulocytes. We found that in contrast to the CD14++CD16+ intermediate monocytes (Additional file 4C), the CD16 positive non-classical (CD14+CD16++) subset exhibited a significant decrease (Additional file 4D) on POD 1 (p < 0.001, N = 24) and a partial recovery by POD 3 (p < 0.001, N = 21). In wound fluid, the subset of non-classical monocytes was essentially absent (data not shown). Thus, the intermediate monocyte population was selectively induced upon liver surgery which may relate to its reported angiogenic capacity and a pertaining potential to promote liver regeneration. While this study has focused on patients with liver resection, a comparable rise in intermediate monocytes to support tissue regeneration might also be envisaged for patients undergoing major surgery other than liver resection. This has, however, not been addressed in the current study.

With respect to the proposed pro-angiogenic TEM subset, there were no substantial fluctuations in the circulating numbers of TEMs after surgery and no correlation with the post-operative release of ANG-2 in patient blood. However, we found an accumulation of TIE2 positive monocytes (CD14++TIE2+ cells) in subhepatic wound fluid which is likely to be conditioned by the regenerating liver. Comparably, our results are in line with preclinical studies which detected TIE2+ monocytes in angiogenic mouse tissue [24]. Rather than the induction of TEMs in blood, the local upregulation of TIE2 expression on monocytes due to environmental factors of the injured tissue might be responsible for this observation. Given the role of TIE2 in angiogenesis, the elevated expression of TIE2 on monocytes would be expected to enhance their angiogenic properties. It has to be noted that this study was designed to unravel monocyte changes and correlations in clinical parameters but did not include functional tests. Hence, the attribution of intermediate monocytes and TEMs as pro-angiogenic subsets relies on the assessment of previous reports [9,20,25].

We further addressed the question whether alterations in the monocyte profile after liver resection are associated with hallmarks of liver injury and regeneration. Therefore, we included liver function parameters from routine hospital evaluation in our analysis. We found positive correlations of intermediate monocyte levels on POD 3 with liver parameters on POD 3–4, whereas measurements from POD 1 did not show significant correlations. While the surge of intermediate monocytes on the first post-operative day is likely an immediate reaction common to all patients, a sustained increase on POD 3 may indeed reflect a delayed, ongoing liver regeneration and reduced organ function as indicated by the elevated levels of liver enzymes on POD 3–4. In line, the median blood levels of angiogenic monocytes on POD 3 tended to be higher in patients with major (17% intermediate subset within CD14++ monocytes) as opposed to minor (8%) surgery (p = 0.065, N = 21), and intermediate monocytes significantly correlated with the level of tissue destruction as evidenced by LDH release. These observations strengthen our conclusion that monocyte populations with proposed angiogenic properties are associated with the process of liver regeneration after surgery. With respect to clinical implications the selective induction of this monocyte subset might therefore be envisioned as a therapeutic approach which merits further investigation: Bearing in mind that postoperative monocyte counts are predictive for overall survival after resection of colorectal liver metastases [7], it has previously been attempted to raise monocyte counts by the perioperative treatment with recombinant GM-CSF [31]. While GM-CSF is known to boost the immunoreactive properties of monocytes [31], it induces the classical rather than the intermediate (CD16+) phenotype [32]. Thus, the short-term perioperative application of M-CSF as opposed to GM-CSF might be more effective in promoting liver regeneration after surgery by selectively enhancing the angiogenic monocyte subsets. Further support to this notion was given by an experimental model of partial hepatectomy in M-CSF deficient mice, where perioperative application of recombinant M-CSF was found to rescue liver regeneration [11].

## Conclusions

In summary, our study provides novel insight into the selective induction of reportedly pro-angiogenic monocyte subsets and regulatory cytokines in response to liver resection; it is the first investigation showing an accumulation of intermediate monocytes and TEMs close to the site of liver regeneration after hepatectomy in a clinical setting. The correlation between these monocyte subsets and liver parameters may indicate a functional link in liver regeneration which warrants further investigation as therapeutic target.

## Additional files

Additional file 1:
**Gating strategy for the detection of monocyte subpopulations by flow cytometry.** **(A)** Leukocytes (P1) were detected in a forward (FS) and side scatter (SS) diagram which comprised lymphocyte (L), monocyte (M) and granulocyte (G) populations. **(B)** CD14-FITC and CD16-PC5 positive cells were then identified among leukocytes. Classical monocytes (CD14++CD16-, gate P2) and intermediate monocytes (CD14++CD16+, gate P3) were defined by their high level of CD14 expression and further discriminated by the presence or absence of CD16 surface marker. Total CD14++ monocyte counts were deduced by combining the measurements of classical CD14++CD16- and intermediate CD14++CD16+ monocytes. **(C)** TIE2 expression (black line) was determined within the total CD14++ monocyte population to detect TEMs (CD14++TIE2+, gate P4) in reference to immunolabelling with mouse IgG_1_-PE isotype control (grey line). Please note that non-classical monocytes (CD14+CD16++) partially overlap with the CD14-CD16++ natural killer (NK) cell subset in their marker profile and were therefore excluded from the analysis. Additional file 2:
**Distribution of monocyte subsets in patients prior to surgery as compared to healthy controls.** Monocyte subsets were determined by flow cytometry in blood samples retrieved from healthy individuals and patients immediately prior to resection of colorectal liver metastases. The frequency of total CD14++ monocytes **(A)** as well as the distribution of classical CD14++CD16- monocytes **(B)**, intermediate CD14++CD16+ monocytes **(C)**, and CD14++TIE2+ TEMs **(D)** were assessed. Additional file 3:
**Liver function parameters and intermediate monocytes do not differ between patients with and without NASH or CASH.** **(A)** Blood levels of bilirubin, **(B)** ASAT, **(C)** ALAT and **(D)** the frequency of intermediate monocytes on post-operative days 3–4 were compared between patients with and without (w/o) NASH or CASH as diagnosed by histopathology of resected liver tissue. Additional file 4:
**Modified gating strategy for perioperative monitoring of non-classical monocytes.** **(A)** A logarithmic leukocyte density plot was introduced to improve resolution of monocytes (M1), lymphocytes (L) and granulocytes (G) based on forward (FS) and side scatter (SS). Setting a tight gate (M1) on the monocyte population enabled us to minimize “contamination” of non-classical monocytes by CD16++ natural killer cells or granulocytes. Classical monocytes (CD14++CD16-, gate M2), intermediate monocytes (CD14++CD16+, gate M3) and non-classical monocytes (CD14+CD16++, gate M4) were then discriminated by their level of CD14 and CD16 expression. The distribution of classical **(B)**, intermediate **(C)**, and non-classical **(D)** subsets within monocytes was evaluated prior to surgery (Pre-OP) and on post-operative days 1 (POD 1) and 3 (POD 3).

## Abbreviations

ALAT: Alanine aminotransferase
ANG-2: Angiopoietin 2
ASAT: Aspartate aminotransferase
CRP: C-reactive protein
CTAD: Citrate, theophylline, adenosine, dipyridamole
EDTA: Ethylenediamine tetraacetic acid
gGT: Gamma-glutamyltransferase
LDH: Lactate dehydrogenase
LPS: Lipopolysaccharide
M-CSF: Macrophage colony stimulating factor
POD: Post-operative day
pre-OP: Pre-operative (prior to surgery)
PT: Prothrombin time
SHW: Subhepatic wound exudate
TEMs: TIE2 expressing monocytes
TGFβ1: Transforming growth factor beta 1
VEGFR2: Vascular endothelial growth factor receptor 2
