# Supplementary figures and images for: Monocytes with angiogenic potential are selectively induced by liver resection and accumulate near the site of liver regeneration
Source: BMC Immunol. 2014 Oct 30;15:50. doi: 10.1186/s12865-014-0050-3 (PMC4223854; doi:10.1186/s12865-014-0050-3)

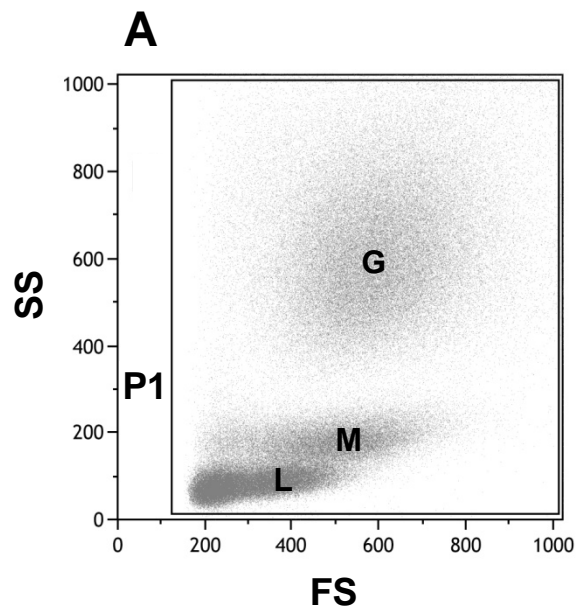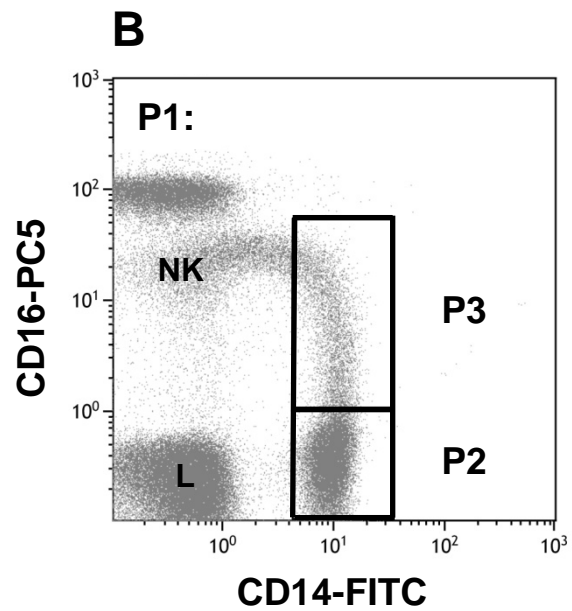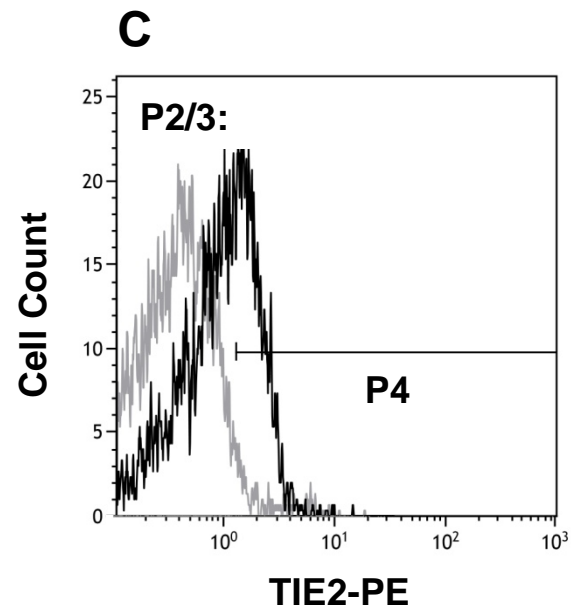

Supplement: Additional file 1: — Gating strategy for the detection of monocyte subpopulations by flow cytometry. (A) Leukocytes (P1) were detected in a forward (FS) and side scatter (SS) diagram which comprised lymphocyte (L), monocyte (M) and granulocyte (G) populations. (B) CD14-FITC and CD16-PC5 positive cells were then identified among leukocytes. Classical monocytes (CD14++CD16-, gate P2) and intermediate monocytes (CD14++CD16+, gate P3) were defined by their high level of CD14 expression and further discriminated by the presence or absence of CD16 surface marker. Total CD14++ monocyte counts were deduced by combining the measurements of classical CD14++CD16- and intermediate CD14++CD16+ monocytes. (C) TIE2 expression (black line) was determined within the total CD14++ monocyte population to detect TEMs (CD14++TIE2+, gate P4) in reference to immunolabelling with mouse IgG1-PE isotype control (grey line). Please note that non-classical monocytes (CD14+CD16++) partially overlap with the CD14-CD16++ natural killer (NK) cell subset in their marker profile and were therefore excluded from the analysis. [file 12865_2014_50_MOESM1_ESM.pdf]

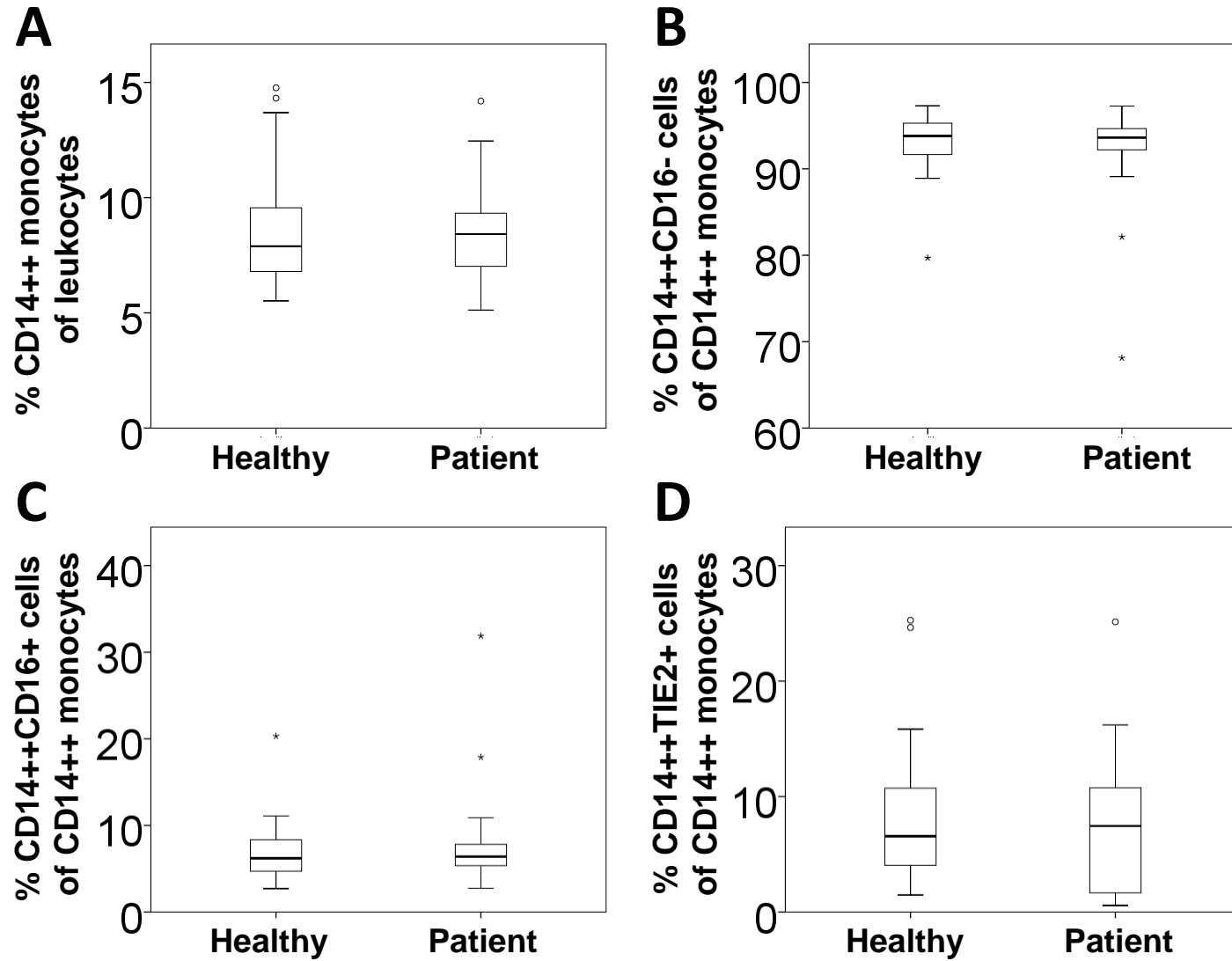

Supplement: Additional file 2: — Distribution of monocyte subsets in patients prior to surgery as compared to healthy controls. Monocyte subsets were determined by flow cytometry in blood samples retrieved from healthy individuals and patients immediately prior to resection of colorectal liver metastases. The frequency of total CD14++ monocytes (A) as well as the distribution of classical CD14++CD16- monocytes (B), intermediate CD14++CD16+ monocytes (C), and CD14++TIE2+ TEMs (D) were assessed. [file 12865_2014_50_MOESM2_ESM.pdf]

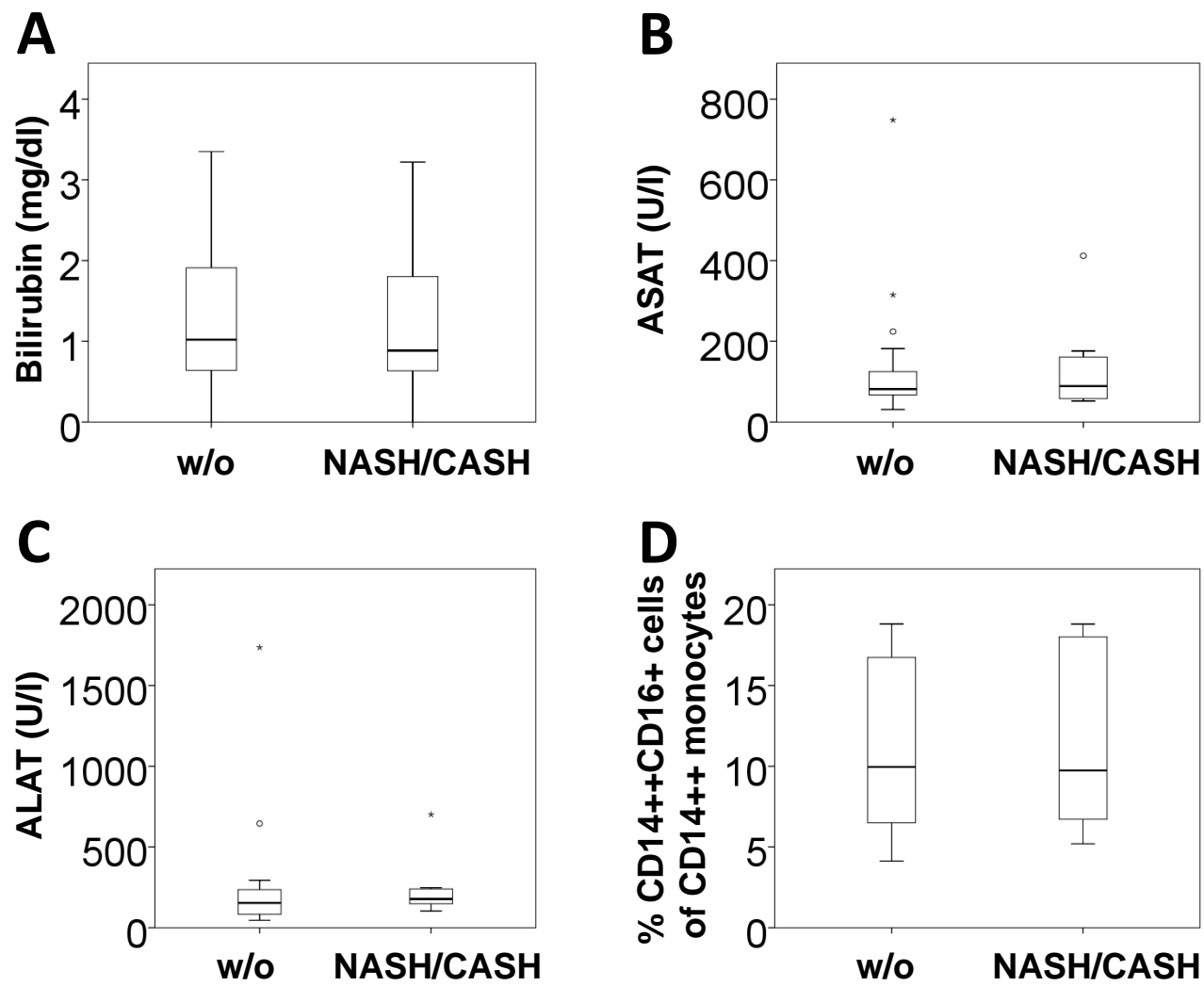

Supplement: Additional file 3: — Liver function parameters and intermediate monocytes do not differ between patients with and without NASH or CASH. (A) Blood levels of bilirubin, (B) ASAT, (C) ALAT and (D) the frequency of intermediate monocytes on post-operative days 3–4 were compared between patients with and without (w/o) NASH or CASH as diagnosed by histopathology of resected liver tissue. [file 12865_2014_50_MOESM3_ESM.pdf]

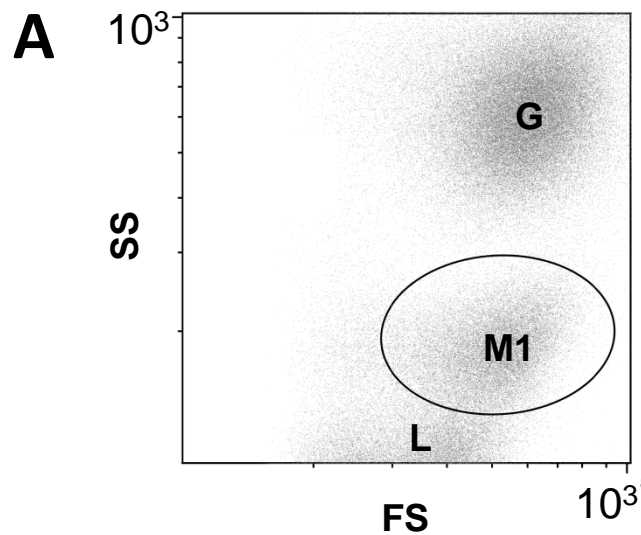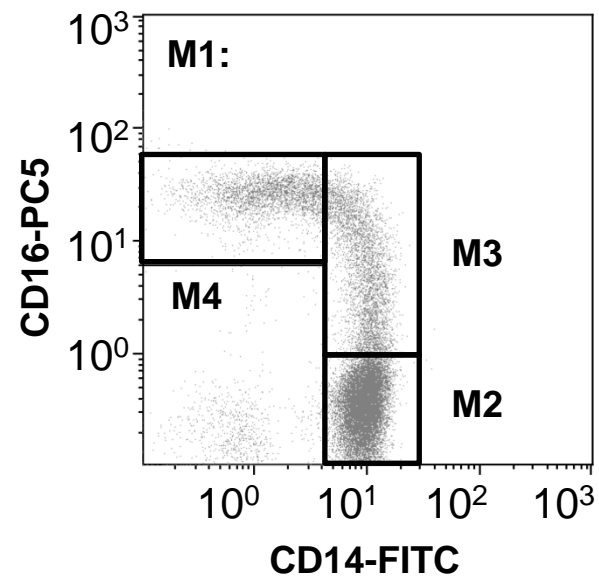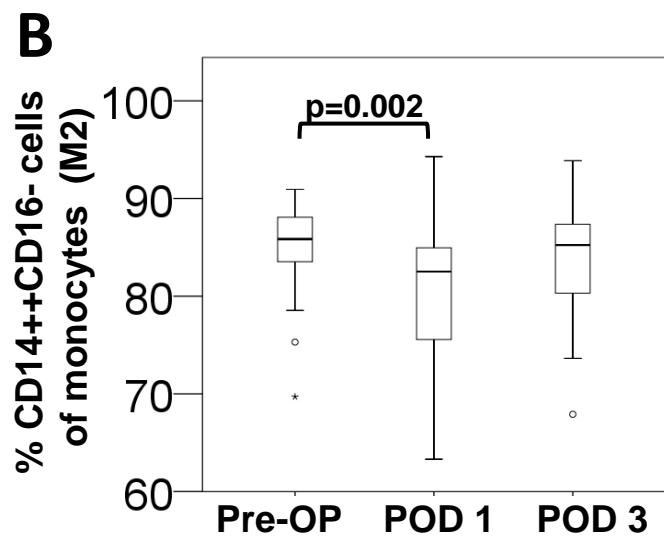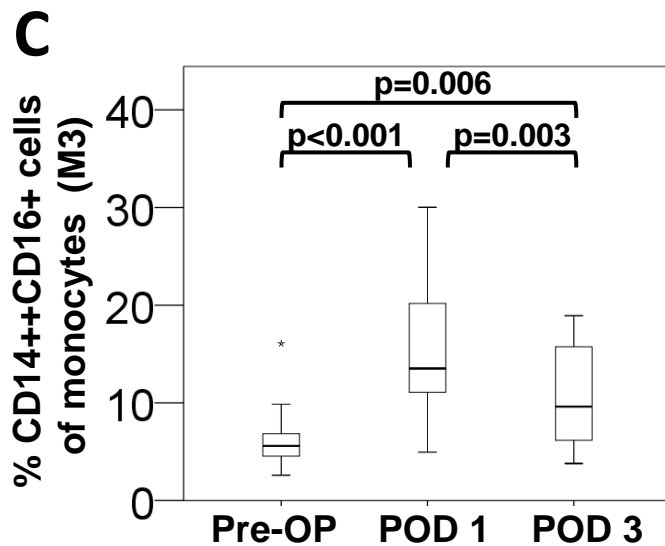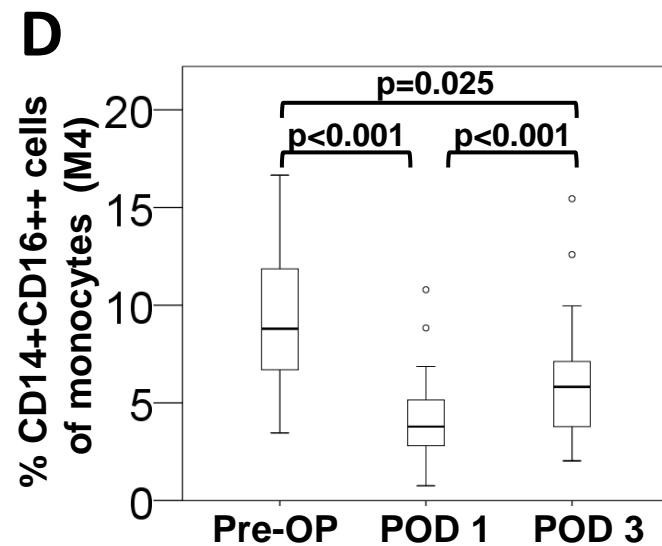

Supplement: Additional file 4: — Modified gating strategy for perioperative monitoring of non-classical monocytes. (A) A logarithmic leukocyte density plot was introduced to improve resolution of monocytes (M1), lymphocytes (L) and granulocytes (G) based on forward (FS) and side scatter (SS). Setting a tight gate (M1) on the monocyte population enabled us to minimize “contamination” of non-classical monocytes by CD16++ natural killer cells or granulocytes. Classical monocytes (CD14++CD16-, gate M2), intermediate monocytes (CD14++CD16+, gate M3) and non-classical monocytes (CD14+CD16++, gate M4) were then discriminated by their level of CD14 and CD16 expression. The distribution of classical (B), intermediate (C), and non-classical (D) subsets within monocytes was evaluated prior to surgery (Pre-OP) and on post-operative days 1 (POD 1) and 3 (POD 3). [file 12865_2014_50_MOESM4_ESM.pdf]
